# The role of AGG interruptions in fragile X repeat expansions: a twenty-year perspective

**DOI:** 10.3389/fgene.2014.00244

**Published:** 2014-07-29

**Authors:** Gary J. Latham, Justine Coppinger, Andrew G. Hadd, Sarah L. Nolin

**Affiliations:** ^1^Asuragen Inc.,Austin, TX, USA; ^2^Department of Human Genetics, New York State Institute for Basic Research in Developmental DisabilitiesStaten Island, NY, USA

**Keywords:** FMR1, fragile X syndrome, repeat expansion diseases, autism spectrum disorders, risk assessment, diagnostic tests

## Abstract

In 1994, it was suggested that AGG interruptions affect the stability of the fragile X triplet repeat. Until recently, however, this hypothesis was not explored on a large scale due primarily to the technical difficulty of determining AGG interruption patterns of the two alleles in females. The recent development of a PCR technology that overcomes this difficulty and accurately identifies the number and position of AGGs has led to several studies that examine their influence on repeat stability. Here, we present a historical perspective of relevant studies published during the last 20 years on AGG interruptions and examine those recent publications that have refined risk estimates for repeat instability and full-mutation expansions.

## INTRODUCTION

Triplet repeat expansions in the 5′ untranslated region of the fragile X gene (*FMR1*) are pathogenic and result in a spectrum of phenotypes, the most well characterized of which is fragile X syndrome (FXS; OMIM #300624, Online Mendelian Inheritance in Man, 2013a). Clinical phenotypes associated with the fragile X premutation span cognitive, behavioral, mood, reproductive, and motor dysfunctions, and include two recognized conditions: fragile X-associated tremor/ataxia syndrome (FXTAS; OMIM #300623, Online Mendelian Inheritance in Man, 2013b) and fragile X-associated primary ovarian insufficiency (FXPOI; OMIM #300624, Online Mendelian Inheritance in Man, 2013a). The complexity of *FMR1-*associated clinical presentation reflects the toxicity of the expanded repeat and the critical role of the protein product, fragile X mental retardation protein (FMRP). FMRP is a selective RNA-binding protein that recognizes defined sequence elements and structures (Darnell et al., 2001, 2011; Ascano et al., 2012) and regulates translational output. FMRP can bind to hundreds of different transcripts to exert translational control. However, its selective regulation of dendritic mRNAs in the brain that are implicated in autism spectrum disorders (ASD) both underlies the frequent diagnosis of ASD in FXS patients and provides a common molecular pathology to link diverse autism phenotypes (Iossifov et al., 2012; Parikshak et al., 2013). In FXS, repeat expansion to greater than 200 CGGs is accompanied by hypermethylation of the repeat region, which shuts down FMRP production and results in a loss of translational regulation by FMRP. In the premutation disorders such as FXTAS, the clinical etiology is thought to be overexpression of *FMR1* mRNA, leading to RNA toxicity and often reductions in FMRP (Hagerman and Hagerman, 2013). Repeat-associated non-AUG translation of *FMR1* mRNA may also contribute to FXTAS (Todd et al., 2013).

## FRAGILE X REPEAT INSTABILITY

The mechanism of fragile X repeat expansion is not well understood, but instability in both mitosis and meiosis is apparent. Mitotic instability is reflected by somatic cell heterogeneity in the number of CGG repeats. Studies that have analyzed size mosaicism in peripheral blood from full-mutation males have demonstrated premutation mosaicism in > 40% of patients (Nolin et al., 1994), and repeat number heterogeneity is evident in distinct tissues from the same individual (Dobkin et al., 1996; Maddalena et al., 1996; MacKenzie et al., 2006).

Meiotic repeat instability is implicated from the observed CGG size difference between parents and children (Nolin et al., 1996), and the variance in repeat numbers in individual sperm cells and lymphocytes in male premutation carriers (Nolin et al., 1999). The specific molecular timing of repeat expansion is unresolved, yet several studies indicate that transmission instability manifests very early in development. Full-mutation, but not premutation, alleles are detected in the ovaries of full-mutation fetuses, suggesting that maternal expansions do not occur purely mitotically in the early embryo from an inherited premutation. In contrast, the testes of a 13-week, but not a 17-week, full-mutation fetus failed to show premutations in the germ cells (Malter et al., 1997) but only premutation alleles are were detected in the sperm of full-mutation males (Reyniers et al., 1993). Since males and females can have categorically distinct fates after transmission, differences in gametogenesis likely contribute to the observed differences in repeat instability from parent to child. Although it is currently unclear how errors in DNA replication (Gerhardt et al., 2014) or repair (Lokanga et al., 2014) may contribute to repeat expansion, *cis* sequence elements can influence the process. The best characterized of these sequence elements is the trinucleotide AGG, which commonly interrupts the CGG repeat tract in normal individuals but has a reduced frequency in carriers.

## AGG INTERRUPTIONS: INCIDENCE, STRUCTURAL IMPLICATIONS, AND DETECTION METHODS

Interrupting AGG sequences were first described in the early 1990s (Eichler et al., 1994). Such interruptions are typically located at the 5′ end of the repeat tract, and are usually arrayed with a periodicity of 9–10 CGG repeats. Approximately 95% of normal individuals have one or two AGG interruptions. Those with a family history of FXS, however, often lack AGGs (Falik-Zaccai et al., 1997).

The low AGG density in longer alleles suggests a role in repeat expansion. Biophysical studies have revealed distinct structures for uninterrupted CGG sequences compared to those with AGG interspersions. For example, oligodeoxynucleotides composed of up to 39 pure CGG repeats form highly stable, stem–loop structures. These hairpins are destabilized by AGG sequences, and different numbers of AGG sequences give rise to distinct conformations (Jarem et al., 2010). As these structures are thought to be key intermediates in trinucleotide instability, it may be that AGG sequences perturb CGG-specific, non B-DNA configurations and thus engage a “biological brake” that curbs expansion.

The idea that non-repeat elements can influence expansion risk was suggested soon after the pathogenic *FMR1* region was identified (Fu et al., 1991; Snow et al., 1993). Eichler et al. (1994) was the first to present evidence that AGG interrupts can reduce the probability of expansion during transmissions from parent to child. A crucial finding from this study was the identification of an “instability threshold” of 34–38 uninterrupted CGG repeats. In the middle to late 1990s, additional studies profiled the AGG structure in both normal and premutation males, and demonstrated: (1) AGG localization to the 5′ repeat segment; (2) CGG sequence variation in the 3′ region; and (3) an increased frequency of uninterrupted CGG stretches in longer sequences (Kunst and Warren, 1994; Zhong et al., 1996).

Until recently, methods to reliably and efficiently interrogate AGG structures from large study cohorts were elusive. Chen et al. (2010) described an accurate PCR-based methodology capable of deducing the AGG interruption pattern in both males and females. This technology resolved the longstanding challenge of untangling signal profiles from multiple, often overlapping, AGG sequences. The assay reports distinct amplicon peaks for each repeat length combination and identifies AGG sequences from characteristic signal losses in the repeat primed CGG trace (“AGG dips”). With capabilities for low DNA inputs, quantitative sizing, single repeat resolution, and a high-throughput workflow, this technology became the method of choice to determine the impact of AGGs upon different facets of *FMR1* biology. The most conspicuous use of this PCR approach has been to assess AGG and CGG genotypes in large study cohorts of allele transmissions from parent to child.

## REFINING THE RISK OF CGG REPEAT EXPANSION WITH AGG INTERRUPTION INFORMATION

Although early studies suggested that repeat expansion was unlikely below a threshold of 50–60 CGGs, it was a mystery why some relatively small expansions were transmitted as full mutations and other larger expansions were stably transmitted. Consequently, expansions were binned into four categories: normal (<45 CGG), intermediate or “gray zone” (45–54 CGG), premutation (55–200 CGG), and full mutation (>200 CGG). These categories were biased by our knowledge of FXS at the time and by our early understanding of expansion risk, which was thought to be limited to those with longer repeats. The more recent emergence of distinct fragile X premutation disorders with a broad spectrum of clinical involvement challenges the ostensible solidity and utility of these longstanding categorical boundaries (Hagerman and Hagerman, 2013). In addition, knowledge gained from AGG mapping studies can now begin to address ambiguities in allele stability that motivated a formal declaration of uncertainty in the so-called “gray zone” or intermediate category.

**FIGURE 1:**
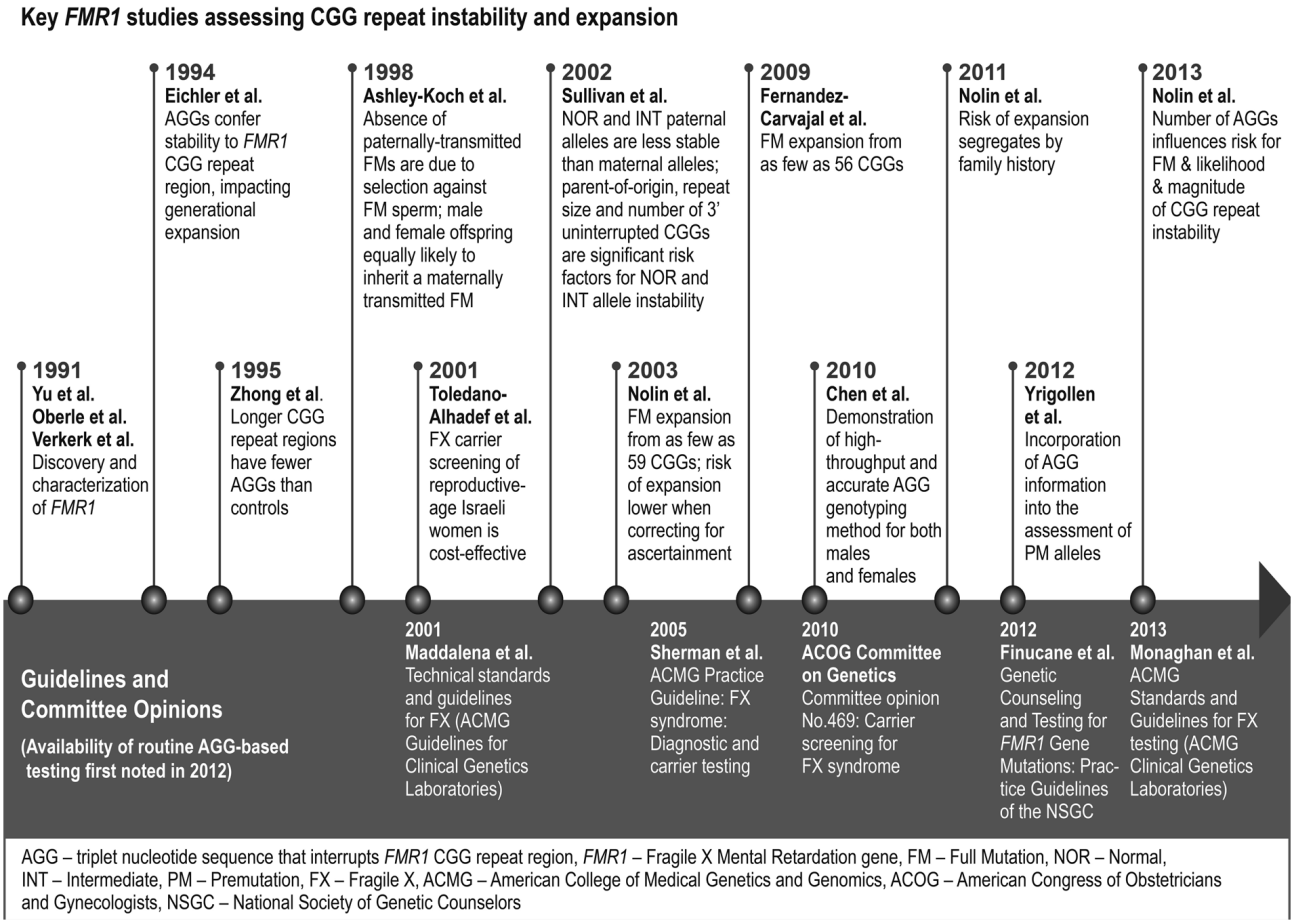
**Timeline for molecular advances and clinical impact of AGG interruptions in the triplet repeat region of the *FMR1* gene.** The figure identifies several of the seminal publications in both basic and clinical research that drove progress in our understanding of the role of AGG interruptions in *FMR1* triplet repeat expansions. Also shown are some of the key professional guideline recommendations and committee opinions relevant to fragile X.

As an understanding of transmission instability came into focus, several groups sought more accurate measures of expansion risk (**Figure 1**). Studies published in the early 1990s evaluated intergenerational stability in families with and without a history of fragile X, and the findings sketched the rough contours of repeat-size determinants (Fu et al., 1991; Snow et al., 1993). Specifically, alleles with < 40 CGGs rarely expanded, alleles with 40–54 CGGs occasionally expanded, and alleles with >54 CGGs commonly expanded—some by hundreds of repeats, causing FXS. These initial reports were extended by Nolin et al. (1996) who assessed the repeat status of 191 families with fragile X and an additional 33 families with gray zone repeats (defined as 40–60 repeats at the time). This study described improved risk statistics for expansion of premutation alleles to full mutations, and for the instability of shorter repeats that did not expand to full mutations. A subsequent study (Nolin et al., 2003) provided updated risk estimates using sample sets and analysis methods that corrected for ascertainment bias. Although the smallest allele that expanded to a full mutation in this cohort was 59 repeats, the overall risk of expansion for alleles with 55–59 CGGs was estimated to be only 1.1–3.7%. By comparison, the full-mutation risk was about 10-fold higher for mothers with 70–79 CGGs, and near 100% for mothers with >100 CGGs. These results (particularly Table 1 of Nolin et al., 2003) served as the basis for more informed genetic counseling of at-risk families for the next decade (Finucane et al., 2012).

A follow-up study of 1112 prenatal samples offered an unbiased cohort that was even more well powered to assess expansion risk as a function of repeat size (Nolin et al., 2011). Overall, the results corroborated previous findings. The study also highlighted the greater risk of families with a history of FXS. For example, 54% of 70–79 CGG alleles from mothers with a family history of FXS expanded to full mutations compared to only 11% with no history. These data pointed to other genetic determinants that influence full-mutation expansions, and AGG interruptions were a leading candidate.

Although these three foundational risk studies (Nolin et al., 1996, 2011, 2003) appraised the impact of total repeat length on transmission instability, each also commented on the potential stabilizing role for AGG interruptions. AGG mapping PCR (Chen et al., 2010) was first utilized in the 2011 Nolin study, and subsequently in a study by Yrigollen et al. (2012) that described a retrospective analysis of AGG interruptions in 267 premutation alleles representing 373 transmissions. As expected, AGG elements substantially impacted the risk of a full-mutation expansion from a given repeat length. For example, the risk varied by 10-fold for 0 (49.6%) versus 2 (4%) AGG at 70 total repeats. Additional factors, such as flanking haplotype markers or maternal age, failed to show statistical significance. The authors noted limitations in the size of the cohort and bias in the population tested, emphasizing the importance of further studies. In addition, the most common repeats in the general population—smaller premutation and intermediate alleles—were not well characterized in this study. Evidence-based risk measures were needed that could help reassure the large fraction of at-risk individuals in this range.

Consequently, Nolin et al. (2013) evaluated AGG interruptions in 457 maternal and 81 paternal transmissions from parents with 45–69 repeats; this repeat range captures 95% of all intermediate and premutation alleles in the population (Seltzer et al., 2012). Unlike Yrigollen et al. (2012), most of the samples were drawn from population screening in this new study. The authors demonstrated that AGG sequences strongly modulated the risk of allele instability: 100/103 of alleles without AGGs were unstable (defined as ≥1 repeat change) compared to 31/159 with 2 AGGs. Remarkably, alleles with 55–59 total repeats but 0–2 AGGs spanned a 19-fold change in instability risk. Consistent with this observation, all 9 of the observed cases of full-mutation expansions in this cohort lacked AGGs. Furthermore, the magnitude of repeat change correlated with the AGG status. The median repeat number change for alleles with 0 AGGs compared to those with 2 AGGs differed by as much as 10-fold (for 65–69 CGGs). Finally, the study demonstrated the greater instability of paternal (81%) compared to maternal (47%) alleles, in agreement with previous reports (Sullivan et al., 2002; Nolin et al., 2011). Loss of AGGs, one plausible mechanism for instability, occurred in a single case, suggesting that this is a rare event.

The seminal publications by Yrigollen et al. (2012) and Nolin et al. (2013) also highlight the challenges in incorporating AGG information into a single mathematical model that can guide patient counseling—arguably the most important practical application of these works. The two studies assessed distinct patient populations, allele distributions, instability measures, and statistical models. Any one of these factors can influence how AGG information is incorporated into a cohesive risk model. Yrigollen et al. (2012) utilized logistic regression and modeling using the Akaike Information Criterion (AIC), which favors goodness of fit with the fewest number of covariables. Nolin et al. (2013) also invoked logistic regression and considered multiple predictor variables, but further used linear regression and ANOVA to assess the magnitude of instability. Both studies identified small differences among the best models that considered various combinations of the AGG number, total repeat length, and the 3′ uninterrupted repeat length. Additional data are needed to refine these models. Nevertheless, it is clear from these two studies, which together represent >900 transmission events, that AGG information can significantly improve risk estimates for individuals with intermediate and premutation alleles. These publications represent practical outcomes from 20 years of research into the role of AGGs in repeat expansion (**Figure 1**), with opportunities to improve genetic counseling and support a broader adoption of fragile X screening.

## IMPLICATIONS OF AGG GENOTYPING IN PATIENT TESTING AND COUNSELING

The mounting evidence from recent risk studies invites discussion for how AGG genotypes may be best incorporated into clinical practice. This discussion, in turn, must consider recent trends in fragile X screening. For example, the American Congress of Obstetricians and Gynecologists (ACOG, 2010) on fragile X carrier screening updated recommendations in 2010 to include women who request screening rather than only those with a fragile X family history. These changes tacitly acknowledge the rapid growth in fragile X carrier screening, as has also been observed for many other genetic disorders. As a result of this trend, we can expect that far greater numbers of women with expanded alleles will be identified in the future.

Large-scale increases in carrier detection present a greater public health need for more informed risk assessment and counseling. The 2013 update to the American College of Medical Genetics (Monaghan et al., 2013) Standards and Guidelines for fragile X testing noted the potential for AGG information to predict expansion risk from premutations with <100 repeats and the availability of direct testing for these sequences (**Figure 1**). However, further study was recommended to determine the clinical usefulness of this testing. These guidelines were finalized with the insight of the Yrigollen et al. (2012) study, but prior to the Nolin et al. (2013) publication. Similarly, the National Society for Genetic Counselors (NSGC) published a revision of practice guidelines in late 2012 (Finucane et al., 2012). Among the updates were recognition of increased carrier screening for women without fragile X risk factors, and the availability of new PCR assays that can help resolve the impact of AGGs in modeling expansion risk. These guidelines also stressed the need to study the impact of AGG information in genetic counseling practice.

The profile of patients that may pursue AGG testing in the future reflects both established high-risk groups and individuals identified by screening. Historically, the assessment of expansion risk has been driven by evidence of family history (including unspecified intellectual disability or ASD) and/or females with ovarian insufficiency. The vast majority of the estimated one million carriers in the US are thus undiagnosed. Several trends, however, are increasing the number of identified carriers. First, carrier screening by academic laboratories and commercial entities using large panels of genes are rapidly gaining momentum. Second, women are increasingly delaying family planning into their third and fourth decades, which elevates the risk of complications and often triggers additional high-complexity testing, including genetic testing. Third, fragile X newborn screening pilot studies have identified individuals with intermediate and premutation alleles; some of these individuals have then been further analyzed using AGG genotyping (Yrigollen et al., 2013).

The majority of those identified with expanded alleles will have intermediate or small premutation alleles with a low *a priori* risk for a child with FXS. AGG testing is expected to provide reassurance to those with AGG interruptions while alerting those with the highest risk for instability. In clinical scenarios such as invasive prenatal testing or assisted reproductive technologies, AGG testing may assist in patient decision-making and medical management. Yet it is well known that other factors besides maternal CGG repeat length and AGG interruptions contribute to the risk of full-mutation expansions (Nolin et al., 2013). The presence of AGGs, though reassuring, does not eliminate this risk altogether. In addition, given that fragile X premutation carriers can have normal phenotypes or a variable collection of clinical and subclinical findings, some of which do not present until adulthood, we anticipate that genetic counseling challenges will arise from the growing number of women identified with modest expansions (for example, 50–54 CGGs) and a quantifiable risk for transmitting a premutation to their offspring.

## FINAL PERSPECTIVES

Over the past two decades, our perception of AGG interruptions has matured from a sequence curiosity to an established stability factor that can help individualize the expansion risk for fragile X carriers. Still, many questions remain at the level of basic and clinical research as well as genetic counseling. How do AGGs impact specific mechanisms of expansion? What effect do they have on genotype–phenotype correlations across different clinical presentations? How should parents be counseled, particularly when their child has a risk to develop a premutation condition? And how does AGG information affect decision making by patients following counseling? These questions each require further study.

The categories initially established for CGG repeat number reflect an understanding of fragile X that has now advanced to recognize clinical presentations beyond canonical FXS. However, our knowledge of the phenotypic spectrum across the full range of CGG repeats is far from complete. Accurate repeat length determination and improved methods for AGG genotyping may influence broader fragile X testing, and help clarify links to phenotype. Instead of relying on normal, intermediate, and premutation allele categories, genetic counselors may incorporate risk factors for expansion into a continuum calculated from repeat length and AGG status. As additional studies seek answers and redefine the fragile X phenotypic spectrum, there is an emerging role for AGG genotyping to clarify the course of fragile X genetic diagnosis, counseling, and patient management.

## Conflict of Interest Statement

G. Latham, J. Coppinger, and A. Hadd are employees of Asuragen and own stock or stock options in the company. G. Latham is an inventor on an issued US patent (8679757) that describes methods for detecting AGG interruptions in the fragile X gene.
